# Polydioxanone Membrane for Guided Conjunctival Tissue Reconstruction: An Experimental Model in Rabbits

**DOI:** 10.1167/tvst.14.5.29

**Published:** 2025-05-28

**Authors:** Daniel Diniz da Gama, Gabriela Martines, Moacyr Rigueiro, Matheus Cruz, Paulo Schor

**Affiliations:** 1 Department of Ophthalmology and Visual Sciences, Paulista School of Medicine, Federal University of São Paulo (UNIFESP), São Paulo-SP, Brazil

**Keywords:** polydioxanone membrane, amniotic membrane, conjunctival reconstruction, ocular surface repair

## Abstract

**Purpose:**

To evaluate the clinical and histopathological performance of polydioxanone (PDO) membranes in conjunctival reconstruction compared with amniotic membrane (AM), assessing epithelialization, inflammation, and tissue integration in a rabbit model.

**Methods:**

Fifteen New Zealand white rabbits underwent conjunctival resection, with each eye receiving either a PDO or AM graft. Animals were euthanized at 7, 14, 21, and 28 days. Clinical and histopathological evaluation included epithelialization, inflammation, fibrosis, granulation tissue, and graft retention.

**Results:**

Both membranes supported conjunctival healing, with no statistically significant differences in epithelialization, inflammation, fibrosis, presence of granulation tissue, or graft remnants. PDO provided structured handling, whereas AM was more delicate but surgically challenging. Histopathology revealed similar inflammatory and regenerative responses, confirming PDO biocompatibility.

**Conclusions:**

The PDO membrane is a viable synthetic alternative to AM for conjunctival reconstruction. Despite minor differences in handling and degradation, PDO exhibited comparable efficacy. Further human studies are needed to validate its application.

**Translational Relevance:**

These findings support the potential use of PDO membranes in ocular surface reconstruction, particularly in settings where AM availability is limited. The use of PDO could expand treatment options for conjunctival defects and enhance surgical outcomes in ophthalmology.

## Introduction

Conditions such as ocular burns, trauma, inflammatory diseases, tumors, and infections can lead to extensive damage to the ocular conjunctiva, requiring surgical reconstruction. The conjunctiva is vital for maintaining ocular health, providing a protective barrier, and ensuring a smooth ocular surface for vision. When this structure is compromised, reconstructive surgery becomes essential to restore its function and integrity. Techniques currently available for ocular surface reconstruction include autologous conjunctival transplantation, oral mucosa transplantation, limbal stem cell transplantation, and grafting with an amniotic membrane (AM).

The amniotic membrane, derived from the innermost layer of the placenta, has emerged as a cornerstone in ocular surface reconstruction due to its unique biochemical and structural properties. AM is rich in bioactive molecules, such as fetal hyaluronic acid, which suppresses transforming growth factor-beta (TGF-β) signaling and inhibits the proliferation and differentiation of limbal and conjunctival fibroblasts into myofibroblasts.^1^^–^^3^ It also promotes the suppression of inflammatory cytokines, including interleukins interleukin-1 alpha (IL-1α), interleukin-1 beta (IL-1β), interleukin-2 (IL-2), interleukin-8 (IL-8), interferon-gamma (IFN-γ), tumor necrosis factor-alpha (TNF-α), fibroblast growth factor (FGF), and platelet-derived growth factor (PDGF).^1^^,^^2^ This combination of properties modulates inflammatory responses, supports tissue healing by suppressing pro-inflammatory cytokines and fibroblast activation, promotes reepithelialization, and ensures effective treatment of complex conjunctival injuries.^3^

The AM has proven effective in promoting corneal epithelial restoration in eyes with persistent epithelial defects caused by corneal ulcers. It has also shown significant benefits in managing acute ocular burns, substantially decreasing scarring and inflammation in conditions such as Stevens–Johnson syndrome, painful bullous keratopathy, symblepharon release, and as a graft for covering blebs in filtering surgeries, including conjunctival erosion caused by Ahmed valve tubes.^4^

Although cryopreservation allows for extended storage, it may alter some biological properties. Cryopreserved amniotic membranes show reduced total protein content and decreased levels of human serum albumin compared to fresh tissue.^5^ Nevertheless, important anti-inflammatory and anti-scarring components such as high molecular weight hyaluronic acid and HC—HA complexes are preserved, maintaining functional activity.^5^ There is also a loss of epithelial cells of the membrane, but it has been observed that the expansion of conjunctival epithelial cells on this condition is greater than in membranes with intact epithelial cells.^1^ The content of the exposed basement membrane of the AM is crucial for cell adhesion, migration, differentiation, and survival due to its rich composition of collagens (types I, III, IV, V, and VI), laminin, fibronectin, and proteoglycans, such as perlecan.^3^ These components act as ligands for cell surface receptors, such as integrins, which mediate cell adhesion and transmit intracellular signals essential for cell migration and differentiation.^3^

However, the widespread use of AM is limited by its dependence on human donation, the need for complex cryopreservation techniques, and its relatively short shelf life. These factors make AM less accessible in many health care settings, especially in regions lacking advanced medical infrastructure. Additionally, as a human-derived tissue, AM poses risks of contamination and transmission of pathogens, despite preservation protocols using antibiotic-rich media, since true sterilization is not possible for cryopreserved AM.

In this context, synthetic alternatives such as the polydioxanone (PDO) membrane, have gained attention. PDO is a fully biodegradable polymer widely used in medical applications, including sutures, tissue engineering (such as bone reconstruction, including orbital) as well as a drug delivery device in ophthalmology.^6^^–^^8^ Its mechanical properties, high biocompatibility, and slow degradation rate make it an attractive candidate for conjunctival reconstruction.

The PDO membrane Plenum Guide (M3 Health Ind. Com. de Prod. Med., Odont. e Correlatos SA, Jundiaí, Sao Paulo, Brazil) presents a surface morphology designed to mimic common extracellular matrix characteristics, with randomly oriented fibers at the submicrometric level, crucial for facilitating cell adhesion and tissue integration, behaving as a histoconductive material and promoting guided tissue regeneration. The membrane demonstrates a slow degradation profile in vivo, compatible with the requirements for sustained support in tissue repair. To the best of our knowledge, the degradation time of PDO on the ocular surface has not yet been specifically studied. However, its behavior in other biomedical contexts—particularly as a suture material—suggests a degradation range from several weeks to up to six months, depending on local tissue conditions.^9^^,^^10^ Additionally, the membrane used in this study had dimensions of 0.05 mm in thickness and 15 mm × 20 mm in area, providing structural integrity while allowing for tissue integration. It has a shelf life of approximately 12 months when maintained between 5°C and 25°C.

Saska et al.^11^ demonstrated through differential scanning calorimetry analysis that the melting temperatures (107.35°C), enthalpy of fusion, and degree of crystallinity of Plenum Guide membranes did not present significant statistical differences before and after the ethylene oxide sterilization process, making its use safe after the process. They also present a tensile strength (σ) of 3.92 ± 0.28 MPa and an elasticity modulus (E) of 30.1 ± 6.25 MPa, with an elongation at break (ε) of 287.96 ± 34.68%. These mechanical properties indicate that the membrane has the necessary strength for the required handling in ophthalmic use, remaining flexible and resistant at the same time. Finally, the membrane was used to stimulate bone regeneration in vivo in rabbits and did not present signs of toxicity.^11^

Despite promising preclinical studies, the role of PDO membranes in ocular surface reconstruction remains largely unexplored. This study aimed to compare the efficacy of PDO and AMs in conjunctival healing in rabbits, focusing on their ability to support epithelialization, reduce inflammation, and integrate with host tissue. By addressing the limitations of AM and evaluating the potential of PDO as a synthetic alternative, this research seeks to contribute to the development of accessible, cost-effective solutions for conjunctival reconstruction.

## Materials and Methods

### Ethical Approval

The study was approved by the Ethics Committee of the Federal University of São Paulo (UNIFESP) under protocol number 1921160222. The experiments complied with the guidelines of the ARVO Best Practices for Using Human Eye Tissue in Research (Nov2021).

### Membrane Acquisition and Preparation

Thirty PDO membranes were donated by Plenum Bioengineering (Jundiaí). These membranes were sterilized with ethylene oxide and stored in sterile packaging at 8°C until use. The AMs were obtained from the tissue bank at the Federal University of São Paulo (UNIFESP, São Paulo-SP, Brazil), cryopreserved at −80°C in a tissue culture medium supplemented with glycerol. The tissue bank does not provide donor-specific information such as age, ethnicity, or medical history regarding the AM used in this study. Before implantation, AMs were thawed, rinsed to remove residual glycerol, and prepared immediately for grafting.

### Experimental Design and Animal Model

Fifteen New Zealand white rabbits (*Oryctolagus cuniculus*), each weighing between 2.0 and 2.5 kg, were used. Both eyes of each rabbit were included in the study, with the right eye serving as a control (AM treated) and the left eye receiving the PDO membrane. This paired design minimized interanimal variability and reduced the total number of animals required, in accordance with ethical guidelines for animal research. All animal procedures in this study adhered to the ARVO Statement for the Use of Animals in Ophthalmic and Vision Research.

The rabbits were randomly assigned to four groups (*n* = 3–4 per group) based on the euthanasia timeline: 7, 14, 21, and 28 days after surgery. This sequential approach allowed the assessment of conjunctival healing and membrane degradation at different time points.

### Surgical Procedures

Under aseptic conditions, each rabbit underwent conjunctival resection under anesthesia. The anesthetic protocol included intramuscular administration of dexmedetomidine hydrochloride (0.25 mg/kg) and topical proximetacaine hydrochloride (5 mg/mL). A 3 × 3 mm section of the bulbar conjunctiva and underlying Tenon's capsule was excised near the limbus, exposing the bare sclera. The defect was immediately covered with either the PDO membrane or AM, both fixated using fibrin adhesive (Beriplast P, Aventis Behring, Hattersheim, Germany).

Postoperative care included daily administration of ciprofloxacin (0.3%) and prednisolone acetate (1%) eye drops two times a day until euthanasia.

### Euthanasia and Tissue Collection

Euthanasia was performed according to the assigned timeline using a two-step protocol: intramuscular administration of dexmedetomidine hydrochloride (1.25 mg/kg) combined with ketamine hydrochloride (30 mg/kg), followed by intravenous injection of thiopental (75 mg/kg). This procedure adhered to the guidelines of the American Veterinary Medical Association.

Immediately after euthanasia, the ocular tissues were collected, fixed in 10% buffered formalin, and processed for histopathological analysis.

### Clinical and Histopathological Evaluation

Each rabbit underwent a clinical evaluation on the day of euthanasia, performed by an experienced examiner blinded to the histopathological results. Clinical parameters assessed included epithelialization (graded 0–3, where 0 = absent; 3 = complete re-epithelialization), fibrosis (graded 0–3, where 0 = absent; 3 = intense fibrosis), inflammation (graded 0–3, where 0 = absent; 3 = intense inflammation), granulation tissue (graded 0–1, where 0 = absent; 1 = present), and graft remnants (graded 0–1, where 0 = absent; 1 = present).

Histopathological analysis followed the same scoring system as the clinical evaluation. Tissue samples were processed via paraffin embedding, sectioned (5 µm thickness), and stained with hematoxylin and eosin. A experienced pathologist, blinded to the clinical assessment, evaluated the same parameters described elsewhere in this article, ensuring an unbiased comparison between the two membranes. To maintain the integrity of the study, the clinical examiner and the pathologist were not informed of each other's evaluations throughout the research process.

### Statistical Analysis

Statistical analyses were conducted using SPSS (IBM, Armonk, NY). The Wilcoxon signed-rank test was used to compare quantitative, nonparametric data between groups. The *Z* test for two proportions was applied to categorical variables. A 95% confidence level (*P* < 0.05) was used to determine statistical significance.

Additionally, a post-hoc power analysis was performed using GPower 3.1.9.4, based on the clinical epithelialization scores observed in this study (AM group: mean 2.40 ± 1.12; PDO group: mean 2.13 ± 1.19; correlation between paired eyes: *r* = 0.784). An effect size of 0.3543 was calculated, classified as small to medium according to Cohen's criteria. Given the sample size of 30 eyes (15 rabbits) and α = 0.05, the statistical power was estimated at 54.37%. An a priori calculation indicated that approximately 59 eyes (30 rabbits) would be required to achieve a power of 80%.

## Results

### Clinical Evaluation

**Figure 1.**
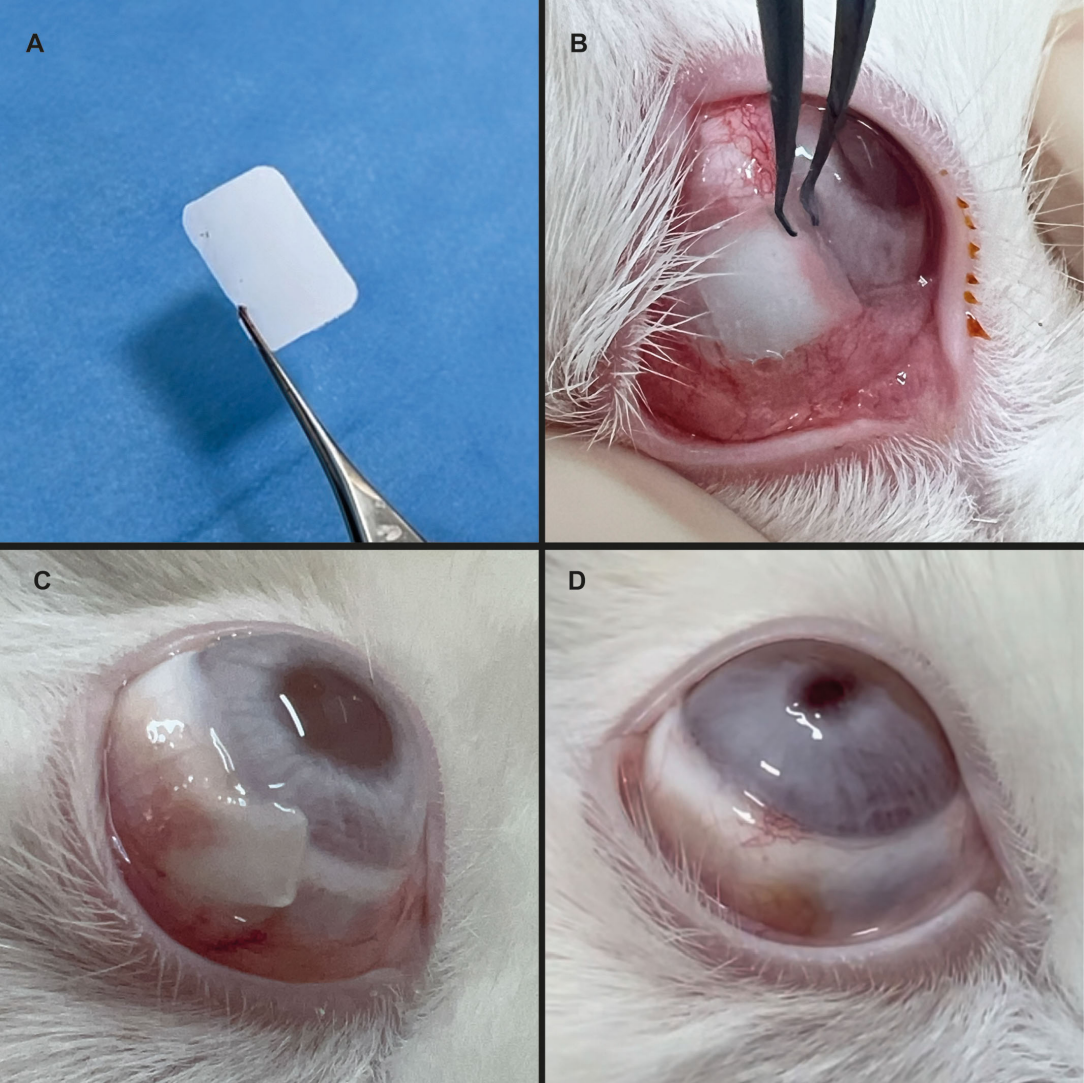
(**A**) PDO membrane (note the rigidity when held with forceps). Examples of the appearance of the PDO membrane at different postoperative stages: (**B**) Immediate postoperative. (**C**) Postoperative day 7. (**D**) Postoperative day 28.

**Figure 2.**
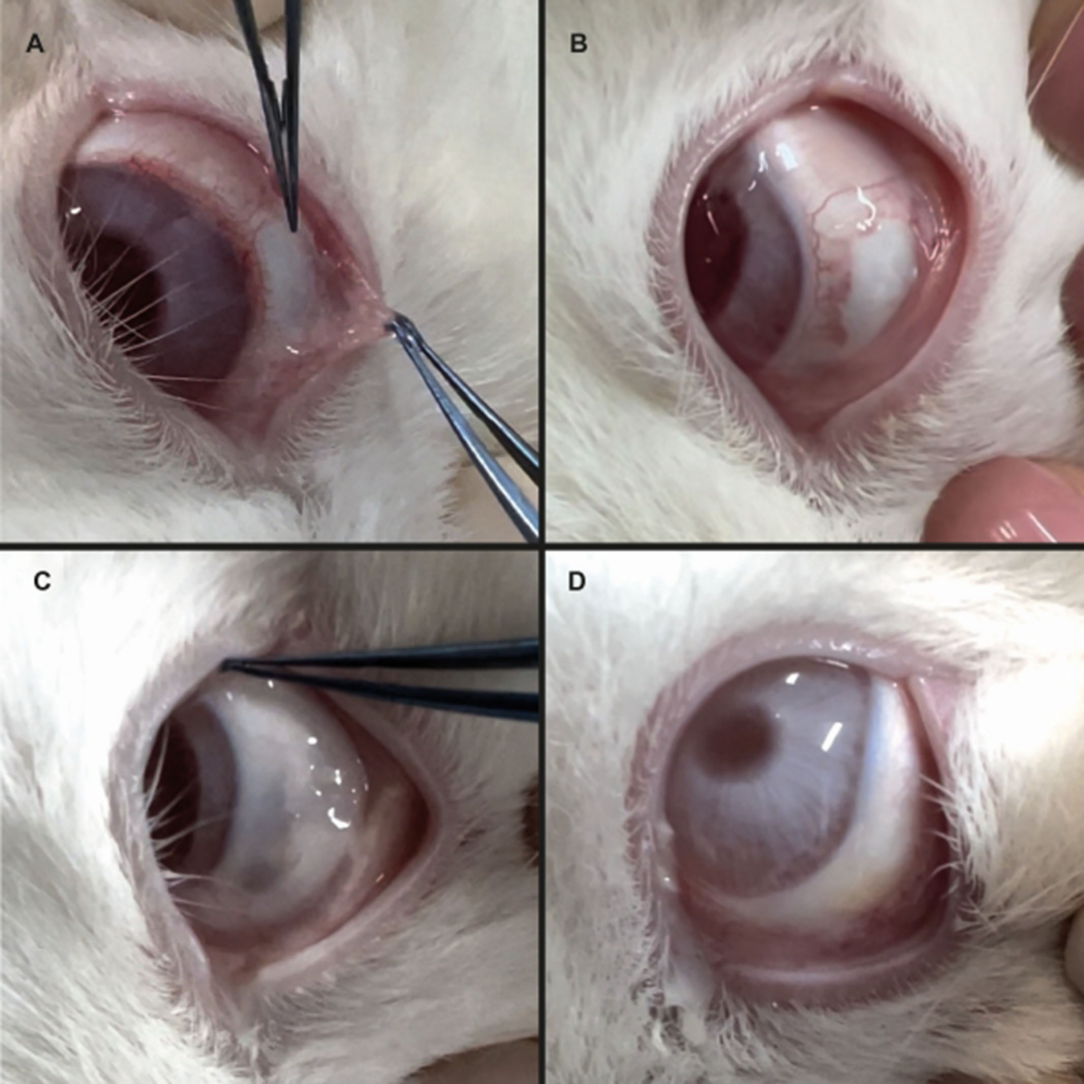
The AM at different postoperative stages. (**A**) Immediate postoperative (notice that it is almost invisible when covering the bare sclera area, identifiable only by its sticky nature when held with forceps). (**B**) Postoperative day 7. (**C**) Postoperative day 21. (**D**) Postoperative day 28.

The clinical assessment of the PDO membrane revealed promising results in conjunctival reconstruction. The membrane demonstrated effective integration with the ocular surface, with visible conjunctivalization occurring as early as postoperative day 7 (Fig. 1). When compared with the AM, the PDO membrane exhibited a more structured handling experience owing to its intrinsic rigidity. In contrast, AM displayed superior mimicry of the natural conjunctival tissue, particularly in terms of transparency and flaccidity, which made it visually more favorable (Fig. 2). However, its gelatinous consistency and adhesive properties, especially on the stromal side, posed challenges during implantation, making precise positioning more difficult.

A key surgical advantage of the PDO membrane was its uniform structure, eliminating the need to distinguish between epithelial and stromal sides during implantation—a notable complexity in AM handling. However, its slightly opaque appearance and increased thickness caused a mild elevation on the ocular surface, which could be perceived as a drawback in terms of tissue homogeneity.

Quantitative clinical evaluation indicated that epithelialization scores were slightly higher for AM (mean, 2.40) compared with the PDO membrane (mean, 2.13; *P* = 0.157). Fibrosis levels were marginally elevated in the PDO group (mean, 0.40) vs. AM (mean, 0.33; *P* = 0.705). Notably, in one case treated with AM, a minor symblepharon was observed. Inflammatory responses were equivalent between the two groups (mean, 0.73; *P* > 0.99).

**Figure 3.**
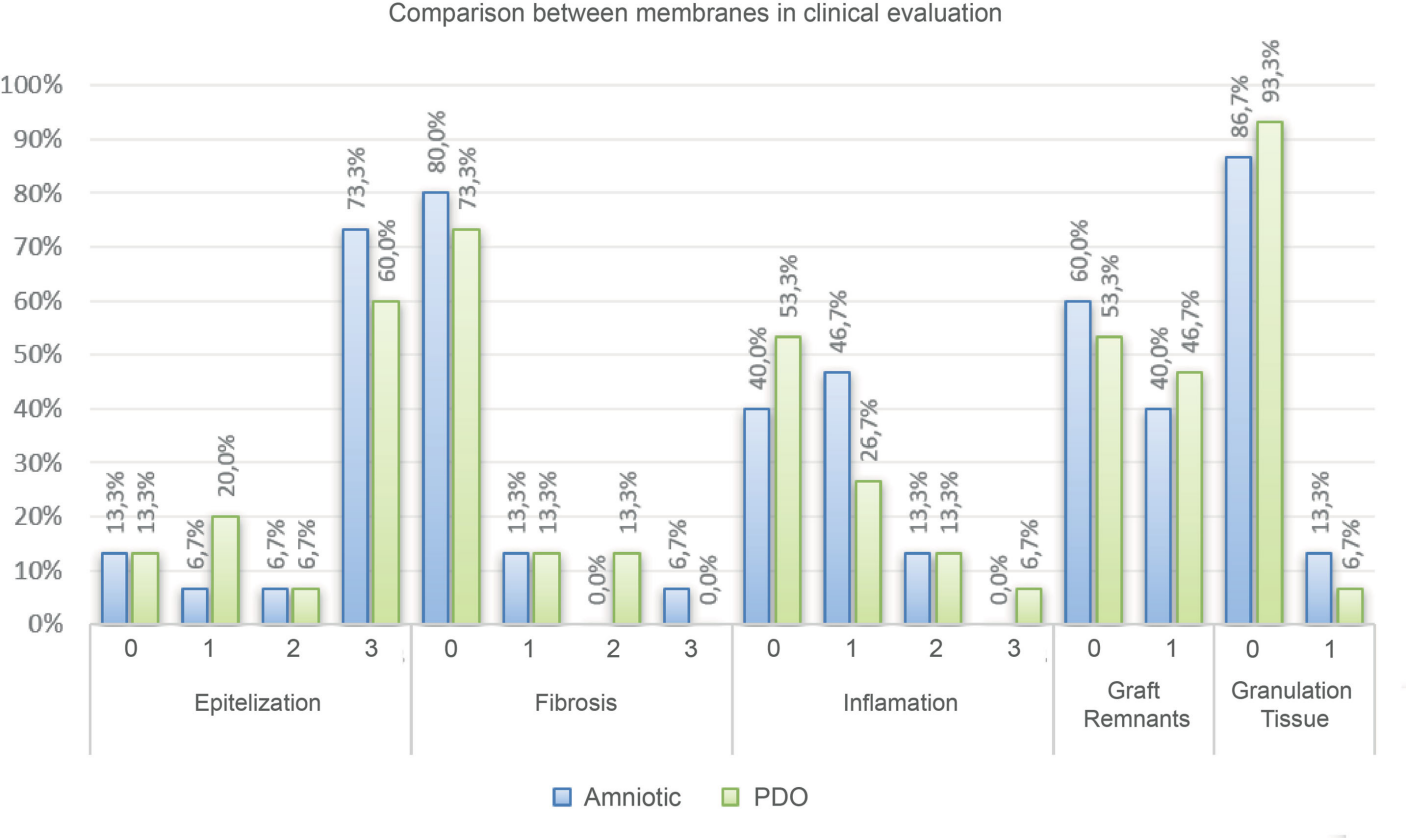
Distribution of scores in clinical evaluation between the membranes.

Granulation tissue formation was slightly lower in PDO-treated eyes (mean, 0.07) compared with AM-treated eyes (mean, 0.13; *P* = 0.564). Graft remnants were observed in 46.7% of PDO-treated eyes and 40% of AM-treated eyes (Fig. 3), indicating a slightly higher retention rate for the synthetic membrane, yet without statistical significance (*P* = 0.739).

In summary, the clinical outcomes revealed no significant differences between the two membranes across the evaluated parameters. Both PDO and AM facilitated successful conjunctival healing without notable complications, suggesting the synthetic PDO membrane as a viable alternative to AM (Table 1).

**Table 1.** Comparison Between Membranes in Clinical Evaluation

	Mean	Median	SD	*N*	CI	*P* Value
Epithelization						
PDO	2.13	3	1.19	15	0.60	0.157
Amniotic	2.40	3	1.12	15	0 .57	
Fibrosis						
PDO	0.40	0	0.74	15	0.37	0.705
Amniotic	0.33	0	0.82	15	0.41	
Inflammation						
PDO	0.73	0	0.96	15	0.49	1.000
Amniotic	0.73	1	0.70	15	0.36	
Granulation tissue						
PDO	0.07	0	0.26	15	0.13	0.564
Amniotic	0.13	0	0.35	15	0.18	
Graft remnants						
PDO	0.47	0	0.52	15	0.26	0.739
Amniotic	0.40	0	0.51	15	0.26	

CI, confidence interval; SD, standard deviation.

### Histopathological Evaluation

**Figure 4.**
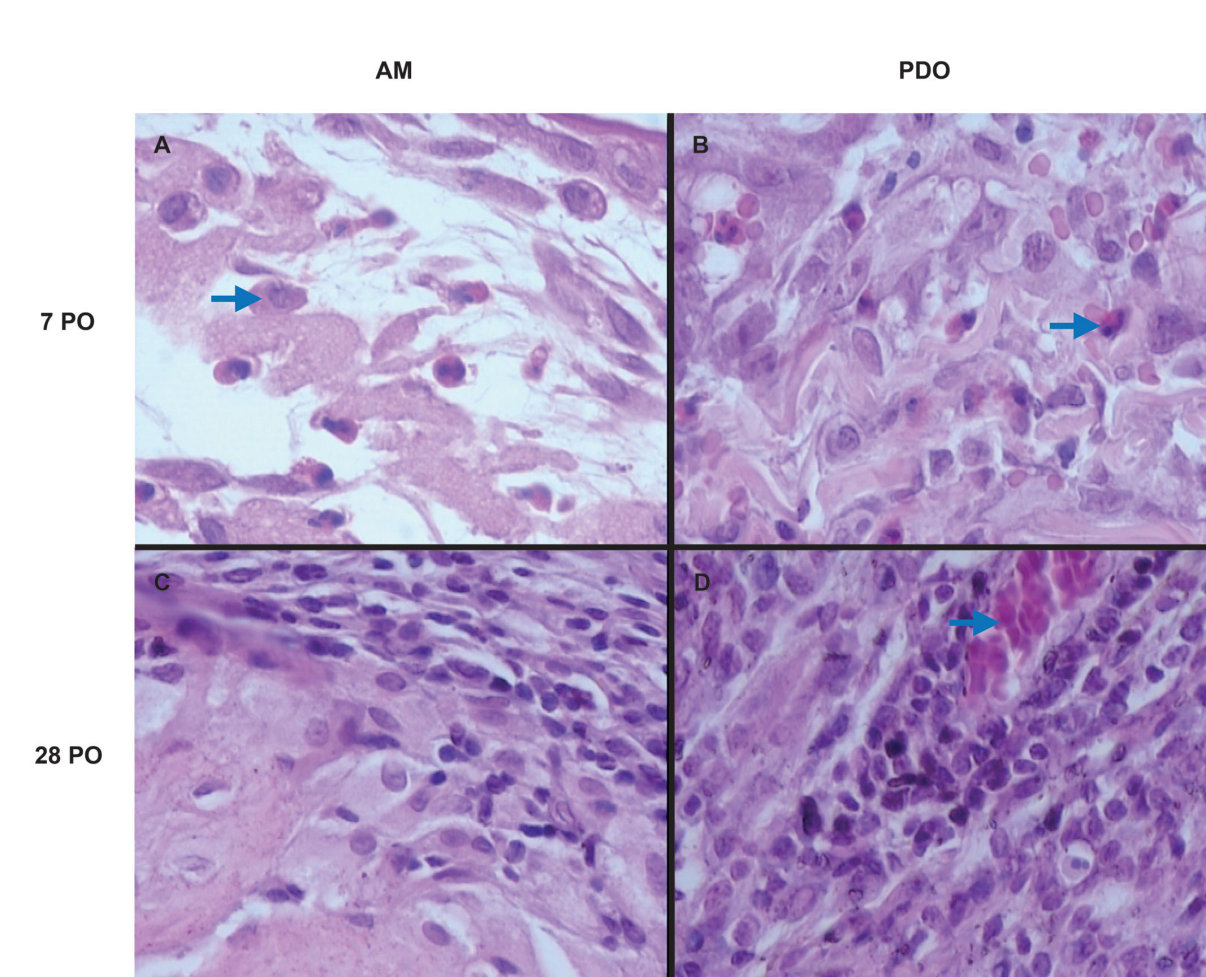
Histopathological findings of the two membranes at different postoperative times at an original magnification of ×400. (**A**) Predominance of macrophages (*arrow*) and lymphocytes, relatively preserved extracellular matrix, and regenerating epithelial cells. (**B**) Predominance of neutrophils (*arrow*) and lymphocytes, extracellular matrix with some areas of tissue disorganization. (**C**) Significant reduction of inflammatory cells, better organization of the extracellular matrix, greater collagen structuring, and mature epithelial cells. (**D**) Moderate presence of inflammatory cells near the graft remnant (*arrow*) with moderate structural disorganization.

**Figure 5.**
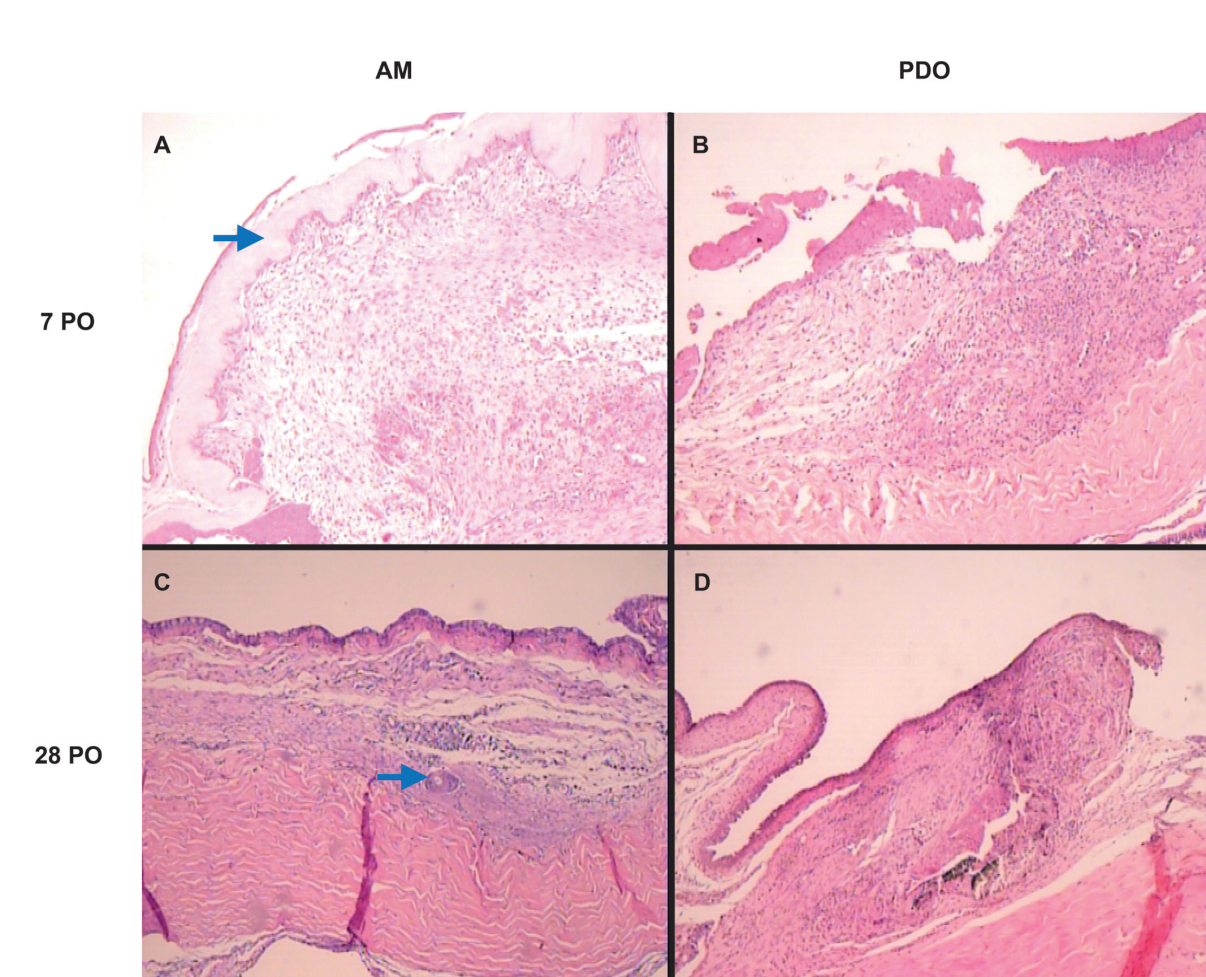
Histopathological findings of the two membranes at different postoperative times at an original magnification of ×40. (**A**) Intense inflammatory reaction around the AM (*arrow*), with good integration of the membrane with the surrounding tissue. The epithelium is immature but nearly complete. (**B**) Less intense inflammatory reaction compared with the AM. In this image, no remnants of the PDO membrane are visible, indicating likely early hydrolysis or resorption. Epithelialization is still incomplete. (**C**) Significantly reduced inflammatory reaction, good tissue integration, and organized extracellular matrix, with complete epithelialization. A foreign body granuloma (*arrow*) is visible. (**D**) Less intense inflammatory reaction, with moderate structural disorganization and epithelialization still with incomplete areas. PO, postoperative (days).

Histopathological analysis further corroborated the clinical findings, highlighting the biocompatibility and regenerative potential of both the PDO and AMs. Notably, inflammatory activity was directly correlated with the persistence of residual graft material. Samples where the PDO membrane was fully absorbed exhibited reduced inflammation and enhanced epithelialization, whereas those retaining graft remnants displayed increased inflammatory cell infiltration, structural disorganization, and granulation tissue formation, even at later postoperative stages (Figs. 4 and 5).

**Figure 6.**
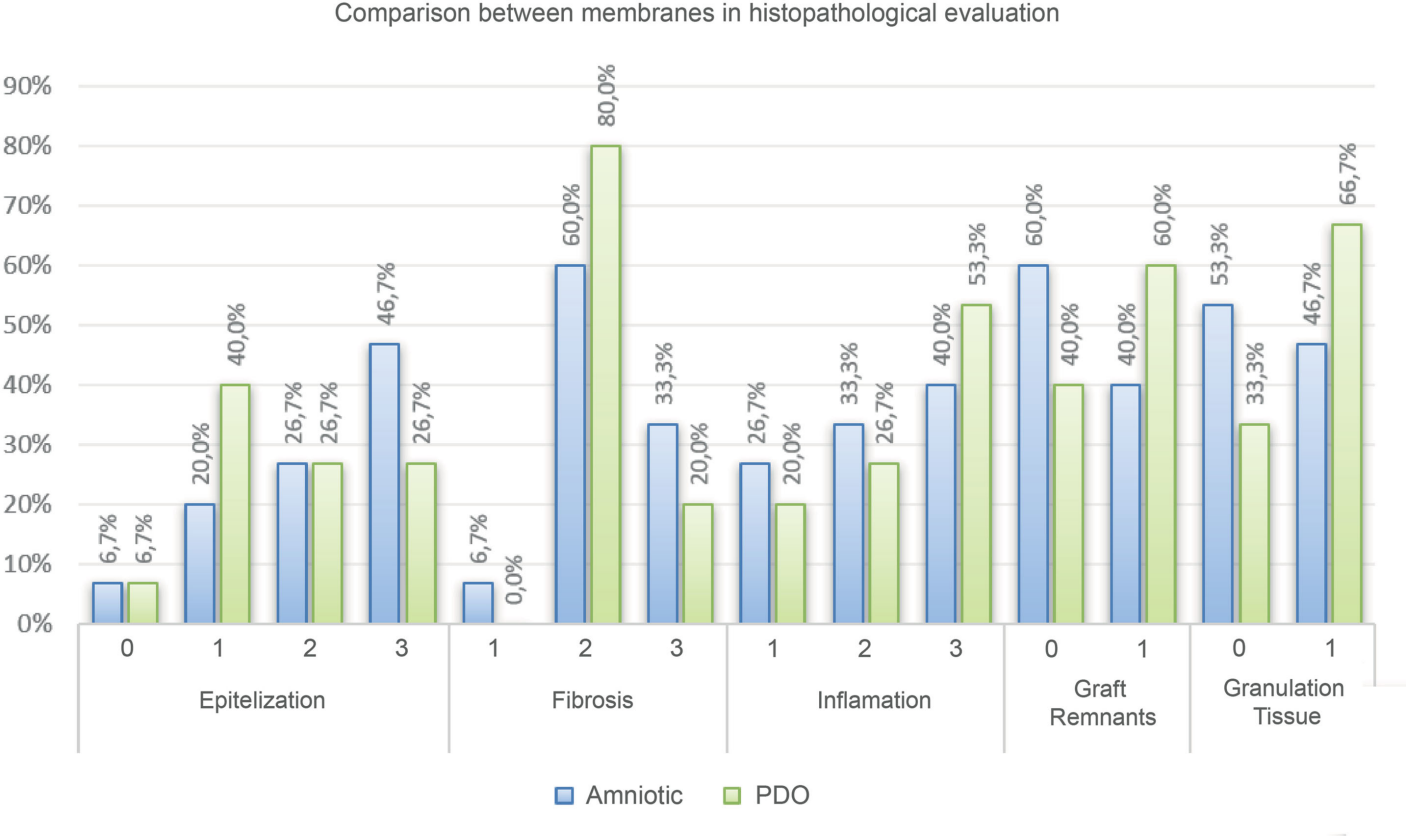
Distribution of scores in histopathological evaluation between the membranes.

Epithelialization was slightly more pronounced in the AM group (mean, 2.13) compared with the PDO group (mean, 1.73; *P* = 0.271). Complete epithelial coverage was achieved in 46.7% of AM-treated cases vs. 26.7% of PDO-treated cases (Fig. 6^7^). Fibrosis was comparable between the two groups, with AM showing a mean fibrosis score of 2.27 vs. 2.20 for PDO (*P* = 0.705).

**Figure 7.**
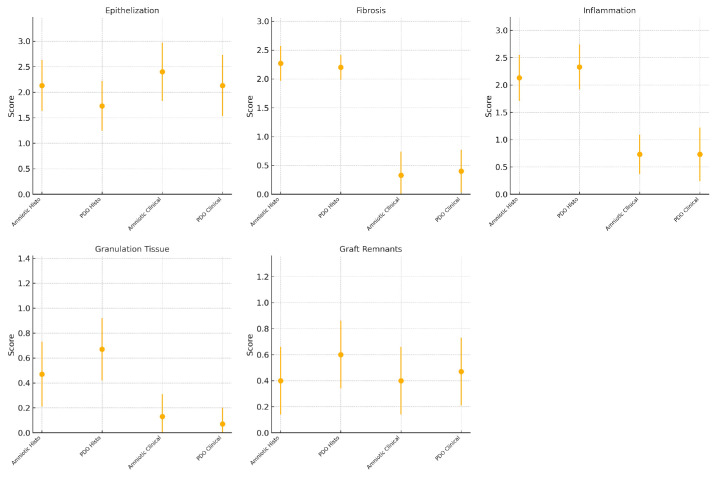
Clinical and histopathological evaluations of the two membranes across the five evaluated criteria (95% confidence interval for the mean).

The inflammatory response was predominantly mononuclear, with a lymphocyte-dominant profile (Fig. 4). Polymorphonuclear cells were primarily localized within granulation tissue foci, and eosinophils were detected in six samples (three from each membrane group), with a maximum of four eosinophils per sample. Inflammatory cells were concentrated in perivascular subepithelial regions or around residual graft material. The overall inflammation score was slightly higher in the PDO group (mean, 2.33) than in the AM group (mean, 2.13; *P* = 0.565).

Granulation tissue formation was slightly increased in the PDO-treated eyes (mean, 0.67) compared with AM-treated eyes (mean, 0.47; *P* = 0.257). The frequency of residual graft material was higher in the PDO group, being observed in 60% of samples, compared with 40% in the AM group (Fig. 6; *P* = 0.257).

The inflammatory cell count per high-power field ranged from 17 to 119 in the AM samples and from 10 to 120 in the PDO samples. The mean inflammatory cell count (Table 2) was slightly greater in AM (61.1 ± 21.3) than in PDO (50.5 ± 17.4; *P* = 0.649). Median values were also comparable (53 for AM vs. 51 for PDO), reinforcing the similar inflammatory response elicited by both membranes.

**Table 2.** Inflammatory Cell Count

	Amniotic	PDO
Mean	61.1	50.5
Median	53	51
SD	42.1	34.4
*N*	15	15
CI	21.3	17.4
*P* value	0.649	

Histopathological evaluation revealed no statistically significant differences between PDO and AM across all assessed parameters (Fig. 7 and Table 3). Although AM demonstrated slightly superior epithelialization, the PDO membrane exhibited similar inflammatory and fibrosis profiles, supporting its potential as a synthetic alternative for conjunctival reconstruction. The observed correlation between graft remnants and inflammation in PDO-treated eyes highlights the need for further refinement of its degradation properties to optimize long-term tissue integration.

**Table 3.** Comparison Between Membranes in Histo-pathological Evaluation

	Mean	Median	SD	*N*	CI	*P* Value
Epithelization						
PDO	1.73	2	0.96	15	0.49	0.271
Amniotic	2.13	2	0.99	15	0.50	
Fibrosis						
PDO	2.20	2	0.41	15	0.21	0.705
Amniotic	2.27	2	0.59	15	0.30	
Inflammation						
PDO	2.33	3	0.82	15	0.41	0.565
Amniotic	2.13	2	0.83	15	0.42	
Granulation tissue						
PDO	0.67	1	0.49	15	0.25	0.257
Amniotic	0.47	0	0.52	15	0.26	
Graft remnants						
PDO	0.60	1	0.51	15	0.26	0.257
Amniotic	0.40	0	0.51	15	0.26	

CI, confidence interval; SD, standard deviation.

## Discussion

The clinical and histopathological results from this study demonstrate that both PDOs and AMs are effective tools for conjunctival reconstruction, with no statistically significant differences in their ability to promote epithelialization, manage inflammation, and prevent fibrosis. These findings highlight the potential of PDO as a synthetic alternative to the AM, particularly in settings where the latter's availability is limited by reliance on human donation, complex cryopreservation processes, and concerns about contamination.

The AM remains a gold standard in ocular surface reconstruction, particularly in large-scale reconstructive surgeries, owing to its unique biochemical composition, including bioactive molecules that suppress inflammation and fibrosis while promoting epithelial migration and differentiation. However, its dependency on human donation limits its widespread application, especially in resource-limited settings. Additionally, its extreme fragility and gelatinous consistency can complicate surgical handling, as also noted in this study.

In contrast, the PDO membrane offers several practical advantages. Its synthetic origin ensures consistent availability and reduces concerns about pathogen transmission. It is also characterized by uniformity and ease of handling. The membrane's rigidity simplifies surgical handling, although its slightly opaque appearance and thickness may pose aesthetic and functional challenges in some cases. While fibrin glue was used in this study, the membrane's structural integrity would also allow for suture fixation, which may be relevant in clinical scenarios where biological adhesives are unavailable or cost-prohibitive. The absence of biological variability in synthetic PDO membranes ensures a consistent product, unlike AM, which can vary depending on donor tissue quality. Furthermore, unlike the amniotic membrane—which requires careful attention to orientation due to distinct stromal and epithelial sides—the PDO membrane has a homogeneous structure, allowing implantation without concern for side orientation, thus simplifying surgical application. Importantly, the slower degradation rate of PDO could be beneficial in complex or extensive conjunctival defects, providing sustained coverage and support during prolonged healing processes. However, this feature may necessitate further optimization to balance degradation time with clinical requirements.

Regarding cost considerations, although one of the proposed advantages of the PDO membrane is the potential reduction of logistical and biological costs associated with the use of amniotic membrane, preliminary market data indicate that the estimated commercial price of the PDO membrane would be approximately USD 130 per unit, compared to an average price of USD 90 per graft for cryopreserved AM in Brazil. It is important to emphasize that this estimated cost of the PDO membrane is based on preliminary projections and may not reflect future market conditions. As the production process is optimized and clinical demand increases, the unit cost of PDO membranes could decrease significantly. This price estimate for the PDO membrane is hypothetical, as it is not yet available in the clinical market for ophthalmologic use, and may reflect manufacturing scale and regulatory costs. Nevertheless, the price difference between the two membranes is relatively small, and the synthetic nature of PDO eliminates costs and logistical demands related to tissue donation, screening, and storage. It is conceivable that, with broader clinical adoption and large-scale production, the cost of PDO membranes could decrease, reinforcing their potential as a standardized and practical alternative to biological membranes, especially in settings where tissue banks are unavailable.

The observed similarity in inflammation and fibrosis rates between the two membranes underscores the biocompatibility of PDO, which did not provoke excessive inflammatory reactions despite lacking the innate anti-inflammatory properties of the AM. The presence of granulation tissue in some PDO-treated eyes highlights the need for further refinement of its physical and biochemical properties to enhance integration and minimize adverse tissue responses.

## Key Takeaways and Future Directions

The findings from this study suggest that the PDO membrane performs comparably with the AM in all evaluated criteria. Despite some minor differences in physical and histopathological aspects, both materials were effective in supporting conjunctival healing and preventing complications such as symblepharon or scleral thinning. The similar epithelialization observed between the two membranes underscores their capacity to effectively guide and support epithelial regeneration, despite their distinct physical and biochemical properties.

The fibrosis noted in both groups highlights the importance of long-term observation to evaluate potential impacts on conjunctival function. It is worth noting that postoperative care challenges in rabbits, including the application of topical medications, might have contributed to higher fibrosis rates in both groups, including the AM, which is widely recognized for its anti-fibrotic properties. This observation suggests that future studies in humans, with more controlled postoperative care, could yield even better outcomes, especially with the AM. Even so, this histopathological finding did not correspond to the clinical evaluation, which observed a harmonious recovery of the ocular conjunctiva, without apparent clinical lesions.

The comparable inflammatory response between the PDO and AMs reflects the high biocompatibility of the PDO membrane, which did not trigger excessive inflammation despite lacking the natural anti-inflammatory components of the AM, such as fetal hyaluronic acid and cytokine-suppressing bioactive molecules.

The presence of granulation tissue, although slightly more pronounced in the PDO group, was not statistically significant, indicating a similar tissue response between the two materials. Furthermore, the primary role of these membranes as scaffolds for epithelialization and protection of the ocular surface appears to outweigh transient inflammatory reactions or structural disorganization observed histologically.

Graft remnants were more frequently observed in the PDO group, although this difference lacked statistical significance. The synthetic nature of the PDO membrane, coupled with its slower degradation profile, might explain this finding. Although a slower absorption rate could benefit more severe or extensive conjunctival injuries by providing prolonged coverage, it might not be ideal for less complex lesions. Future studies should explore modifications to the membrane's degradation rate to optimize its clinical application.

Future research should include clinical trials in human subjects to validate these findings and address the limitations of the current study, such as the small sample size and reliance on an animal model. Although the sample size was adequate for an animal trial, it may have limited the statistical power to detect smaller differences between groups. Ethical considerations and logistical constraints limited the number of animals used, which should be taken into account when interpreting the findings. Additionally, exploring modifications to the PDO membrane, such as incorporating bioactive molecules or adjusting its degradation profile, could further enhance its performance and broaden its applicability. Future comparative cost analyses and long-term follow-up studies will also be essential to determine the economic and clinical feasibility of replacing or complementing the AM with synthetic alternatives like PDO.

In conclusion, this study provides compelling evidence supporting the use of PDO membranes as a synthetic alternative for conjunctival reconstruction. By addressing the limitations of the AM and demonstrating comparable efficacy, PDO represents a promising step toward more accessible and cost-effective solutions for ocular surface repair in diverse health care settings.
